# Inflammatory periosteal reaction on ribs associated with lower respiratory tract disease: A method for recording prevalence from sites with differing preservation

**DOI:** 10.1002/ajpa.23769

**Published:** 2019-01-05

**Authors:** Anna M. Davies‐Barrett, Daniel Antoine, Charlotte A. Roberts

**Affiliations:** ^1^ Department of Ancient Egypt and Sudan The British Museum London United Kingdom; ^2^ Department of Archaeology Durham University Durham United Kingdom

**Keywords:** Middle Nile Valley, new bone formation, pleural disease, prevalence, rib lesions

## Abstract

**Objectives:**

Inflammatory periosteal reaction (IPR) on the visceral surfaces of the ribs has been used in bioarchaeology as an indicator of lower respiratory tract disease. This article presents a detailed method for recording IPR on the ribs, even those in severely fragmented states, with the objectives of increasing the consistency of recording and producing true prevalence rates for skeletons so as to improve data comparability between future bioarchaeological studies of lower respiratory tract disease.

**Materials and methods:**

The presence and prevalence of respiratory‐related IPR were recorded from three different Sudanese cemetery sites using a detailed method for identifying and recording IPR. Sites with variable preservation were chosen to test the applicability of the method. A flowchart to aid in identification of bony changes is presented. The method requires the recording of IPR on three separate sections of the rib (neck, angle, and shaft) and the allocation of ribs into rib cage regions of upper, upper‐middle, middle, lower‐middle, and lower.

**Results:**

Results demonstrate differences in the distribution of IPR between sites and verify the method's applicability to archeological sites with various levels of skeletal preservation.

**Discussion:**

While crude prevalence rates can indicate the number of individuals experiencing lower respiratory tract disease within a site, this method can provide information about the distribution of IPR within the rib cage. This should lead to new ways of distinguishing respiratory diseases within archeological populations. This method also allows for comparability between well‐preserved and lesser‐preserved sites by accommodating for rib fragmentation.

## 1 INTRODUCTION

The presence of periosteal reaction, producing new bone on the cortical layer of the visceral (inner) surface of the ribs, from archeological skeletons has been used as evidence for lower respiratory tract disease in the archeological record. One of the main causes of periosteal reaction on any bone is considered to be infection‐induced inflammation of the surrounding tissue, although other stimuli such as trauma, hemorrhage, and metabolic disease may also be responsible (Ortner, 2003, p. 88; Waldron, 2009, Table 6.7; Weston, 2012). The pleural cavity, a thin membrane‐lined space, lies directly between the lungs and the visceral surfaces of the ribs (Light, 2013, p. 1). Consequently, it has been proposed that fluid or pus accumulation and inflammation within the pleural cavity, commonly caused by lower respiratory tract diseases, may in turn stimulate inflammatory periosteal reaction (IPR) on the visceral surfaces of the ribs (Kelley & Micozzi, 1984; Pfeiffer, 1991; Roberts, Boylston, Buckley, Chamberlain, & Murphy, 1998; Roberts, Lucy, & Manchester, 1994). In clinical contexts, the most common respiratory infections to cause pleural inflammation include tuberculosis, pneumonia, and actinomycosis (Kass, Williams, & Reamy, 2007).

Clinically, thickening of the ribs in radiographs, assumed to be chronic periosteal reaction leading to new bone formation, has been noted in patients suffering from pleural disease (Eyler et al., 1996; Guttentag & Salwen, 1999). Using radiographs, Eyler et al. (1996) investigated the difference in the thickness of ribs in patients with unilateral tuberculosis or empyema (pus accumulation in the pleural cavity) and found that ribs were significantly thicker on the diseased side of the thorax when compared to the non‐diseased side. It was hypothesized that hyperemia (increased blood flow to a specific region) due to an adjacent inflammatory process was responsible for the increase in thickness. Additionally, heart failure, pulmonary embolism, and malignancy such as mesothelioma, among other less common diseases, can also cause fluid accumulation and potential inflammation of the pleura (Light, 2013, p. 128; Porcel & Light, 2006). This may also be considered a differential diagnosis for IPR on ribs.

Studies of recent documented skeletal remains with associated biographical information for each individual, including cause of death, have found a significant association between IPR on the visceral surfaces of the ribs and pulmonary tuberculosis (Matos & Santos, 2006; Roberts et al., 1994; Santos & Roberts, 2001, 2006). However, in these studies, IPR was also observed in 15–36% of individuals dying from other pulmonary diseases, including pneumonia, bronchitis, emphysema, and pleurisy. Unfortunately, individuals from documented collections with cause‐of‐death data—which may or may not be correct—often have limited medical records that do not give an overview of the health problems they experienced throughout their lives. They may also have had co‐morbidities at death that are not mentioned in the cause of death records (Matos & Santos, 2006; Roberts, Boylston, et al., 1998; Santos & Roberts, 2001). Bioarchaeologists are limited to looking at skeletons at the time of death and cannot necessarily trace all health problems experienced throughout the lives of these people.

Kelley and Micozzi (1984) produced one of the first studies of the specific causes of rib IPR, using the Hamann–Todd Collection (Natural History Museum, Cleveland, OH). This study selected individuals who were recorded to have died from some form of tuberculosis or pneumonia. While the side and the rib number affected by IPR or destructive lesions were recorded and it was noted that the central rib shaft tended to be involved more than the head or neck, prevalence rates for lesions on different regions of the rib cage were not presented. Additionally, the lack of investigation into the prevalence of rib lesions within the entire collection, rather than those individuals just with a cause of death of tuberculosis or pneumonia, did not consider alternative causes for these lesions (Roberts, 1999). Later, Roberts et al. (1994) investigated the frequency of rib IPR and destruction in all individuals with complete rib cages from the Roberts J. Terry Collection (National Museum of Natural History, Smithsonian Institution, Washington DC). As well as presenting frequencies according to rib number and rib side, this study recorded the prevalence of lesions on the head, neck, angle and anterior portion of the shaft, as well as in the upper (ribs 1–4), middle (ribs 5–8), and lower (ribs 9–12) regions of the rib cage. Differences were found between the cause of death (either tuberculosis or non‐pulmonary) and the section of rib or the region of the rib cage affected. Further studies on the Coimbra Identified Skeletal Collection (Department of Anthropology, University of Coimbra) (Santos & Roberts, 2001, 2006) and the Human Identified Collection curated by the Museu Bocage, in Lisbon (Matos & Santos, 2006), both in Portugal, and the identified skeletal collection from the Certosa graveyard in Bologna, Italy (Anthropology Museum, University of Bologna) (Mariotti et al., 2015) have followed similar methods. Although these studies have recorded the frequency of rib lesions on the neck, body and sternal end, only Matos and Santos (2006) have provided prevalence rates for the sections of the rib affected. It should be noted that these methods were developed on relatively well‐preserved collections. Preservation of human skeletal remains recovered from archeological sites can be far more variable.

Clinical studies have also shown that particulate pollution within the air causes irritation and inflammation of the respiratory tract (Lee, Kinney, Chillrud, & Jack, 2015; Morales‐Bárcenas et al., 2015). Sustained exposure to particulate pollution, for example, during activities related to certain occupations that produce particulate matter, can cause interstitial lung disease, resulting in chronic inflammation and fibrosis of the lungs (Gold et al., 2000; Rom, Bitterman, Rennard, Cantin, & Crystal, 1987; Ross & Murray, 2004; Sirajuddin & Kanne, 2009; Sood, 2012). This in turn may produce IPR on the ribs. Damage to the lungs caused by particulate matter also increases susceptibility to infectious pulmonary diseases (Rylance et al., 2015; van Eeden et al., 2001). Particulate pollution is of increasing concern today and is estimated to cause 4.2 million premature deaths around the world (World Health Organization, 2018). Thus, investigations of lower respiratory tract disease in past populations can provide a valuable historical context to this modern problem.

In archeological skeletons, the prevalence of IPR on ribs may offer useful insight into the presence of, and exposure to, lower respiratory tract diseases, as well as provide information about poor air quality from many causes. For example, cramped and badly ventilated living quarters and exposure to particulate pollution such as dust, sand, pollen, cooking fires, and habitual smoking have all been discussed as causative factors in relation to the presence of IPR in different archeological settings, and high population density also predisposes to the ready transmission of lower respiratory tract diseases (Binder, 2014, pp. 303–305; Capasso, 2000; Lambert, 2002; Pfeiffer, 1991; Roberts, 2007; Walker & Henderson, 2010). Therefore, accurate recording of IPR may be a valuable tool in investigating evidence of specific respiratory diseases and associated risk factors for respiratory inflammation in past living environments.

Within bioarchaeological studies, the prevalence of a disease has often been presented as the percentage of individuals displaying pathological changes associated with the disease within the sample group studied, referred to as the crude prevalence (Klaus, 2014; Waldron, 2007, p. 62). However, this method does not always take into account the preservation and observability of the specific skeletal elements that manifest the pathological changes (Dutour, 2008, p.133). By recording the presence of a specific skeletal element within an entire sample group and calculating the percentage of those bone elements observed and affected by pathological changes, it is possible to present a more appropriate prevalence rate. This is often referred to as true prevalence. This can be taken further by presenting prevalence according to observable sites on one bone element. For example, to adjust for variability in surface preservation and wear in teeth, Hillson (2001) proposed a method of recording dental caries in which the prevalence for each observable surface of each tooth type is presented separately.

The prevalence of IPR has been presented in various ways in palaeopathological studies, usually as a crude prevalence rate (Bernofsky, 2010; Lambert, 2002; Roberts, Lewis, & Boocock, 1998). In well preserved skeletal remains, the frequency of each of the 12 numbered ribs displaying IPR has also been presented, but not with associated prevalence (Kelley & Micozzi, 1984; Kelley, Murphy, Levesque, & Sledzik, 1994; Matos & Santos, 2006; Santos & Roberts, 2001). It is likely that the region of the rib cage affected (e.g., upper, middle, or lower rib cage) and specific areas of individual ribs (e.g., neck, angle, and shaft) may provide insights as to the type of disease leading to IPR (Santos & Roberts, 2006; Waldron, 2009, p. 117). Although some researchers have noted the location and position of new bone formation affecting the rib cage, and the individual ribs themselves, only Roberts et al. (1994) and Matos and Santos (2006) have presented prevalence rates for the specific parts of the ribs affected, and only Roberts et al. (1994) has published prevalence rates for different regions of the rib cage. Additionally, the criteria used to determine frequencies for different sections of the rib have not been standardized or their morphological delimitations clearly defined by previous studies, making it difficult to compare results between studies. The development of methods on well preserved collections (Kelley & Micozzi, 1984; Roberts et al., 1994) and the lack of a standardized recording method for establishing the true prevalence rate of IPR and its distribution across the rib cage in different archeological skeletal remains has probably hindered interpretations and limited investigations into the potential identity of causative diseases.

In particular, the poor preservation of the ribs in archeological skeletons affects the proper identification and recording of the areas affected by IPR (Roberts, Boylston, et al., 1998; Santos & Roberts, 2006), often resulting in poorly preserved skeletons or whole sites being overlooked (Brickley & Buckberry, 2015). The survival of different sections of the rib is often variable, with the neck and angle usually more likely to survive intact than the main shaft. Thus, post‐mortem fragmentation often prevents observation of certain regions of the rib cage. This certainly affects the comparability of results within and between studies when presenting prevalence according to the number of individuals within the sample affected. A study presenting crude prevalence of IPR from skeletons from a well preserved site is not comparable to a study presenting crude prevalence from a poorly preserved site, where observation of only a small portion of the rib cage of each individual is possible.

Here, a detailed method for recording IPR on the visceral surfaces of the ribs is presented, with the objectives of increasing the consistency of recording and producing true prevalence rates for lower respiratory tract disease in skeletons with widely differing preservation. The ultimate goal is to improve data comparability between future bioarchaeological studies of lower respiratory tract disease, allowing palaeopathologists to apply more rigor in their differential diagnoses.

## 2 MATERIALS AND METHODS

### 2.1 Materials

**Figure 1:**
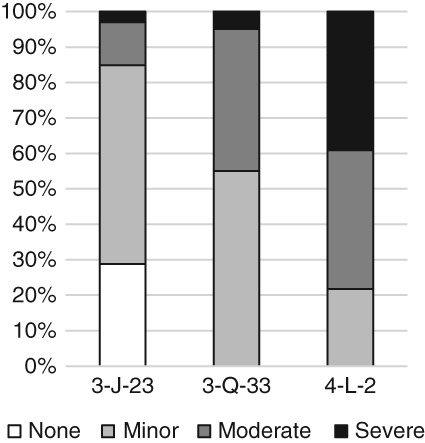
Proportion of skeletons from the sites of 3‐J‐23, 3‐Q‐33, and 4‐L‐2 with rib cage fragmentation scores of none (0), minor (1), moderate (2), and severe (3)

**Figure 2:**
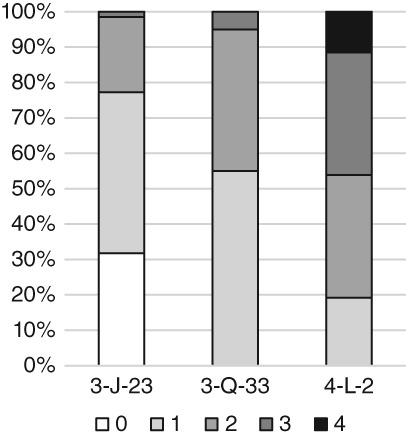
Proportion of skeletons from the sites of 3‐J‐23, 3‐Q‐33, and 4‐L‐2 with rib cage cortical preservation scores of 0, 1, 2, 3, or 4

The diagnostic criteria and methods described below for recording true prevalence rates of IPR were applied to skeletons from three archeological sites curated by The British Museum, London. Normal growth of juvenile bone can have the same disorganized appearance as woven bone caused by pathological stimuli, and can lead to confusion in the identification of IPR (Lewis, 2017, p. 132; Ortner, 2012), particularly at the vertebral and sternal ends of the ribs. Therefore, this study is limited to only adults and adolescents in the latter stages of skeletal development. Skeletons were only included if at least one of the costo‐vertebral facets of the ribs had begun to fuse, which typically occurs after the age of 17 years (Scheuer & Black, 2000, p. 242). This research is part of a broader study of Middle Nile valley populations that includes 12 archeological sites ranging from the Neolithic to the medieval periods. The method was developed to allow comparisons between skeletons from sites with differing preservation. The sites presented here were chosen to represent the range of preservation states encountered in the study, including different levels of rib fragmentation (Figure 1) and cortical preservation (Figure 2), to allow for the testing of the applicability of this method to the rib cages of skeletons with varying degrees of preservation. All sites are located in the Fourth Cataract of Sudan, in the Middle Nile Valley.

The site of 3‐J‐23 consists of 66 individuals dating to the Medieval period in Sudan (c. AD 500–1,500), 20 individuals from the site of 3‐Q‐33 date to the Meroitic and Post‐Meroitic periods (c. 300 BC–AD 600), and 26 individuals from the site of 4‐L‐2 date to the Kerma Classique period (1,750–1,500 BC). 3‐J‐23 presents a low mean rib cage fragmentation score of 0.9; 3‐Q‐33 (1.5) and 4‐L‐2 (2.3) have higher fragmentation scores. The mean cortical preservation score at 3‐J‐23 is also low (0.9), with 3‐Q‐33 at 1.5 and 4‐L‐2 at 2.4 showing some surface degradation (see below for fragmentation and cortical preservation scoring methods).

### 2.2 Fragmentation and cortical surface preservation

The suitability of the method was tested using skeletons in various states of fragmentation, with the rib cage of each individual attributed a fragmentation score of either “none” (0), “minor” [1], “moderate” [2], or “severe” (3) (Table 1), based on the overall percentage of the intact rib cage and the size and number of fragments. These scores were developed from similar methods used for scoring fragmentation or completeness of skeletal remains (Behrensmeyer, 1978; Bello, Thomann, Signoli, Dutour, & Andrews, 2006; Judd, 2001), and were produced to provide only a general comparison of the fragmentation between skeletons from different sites. Cortical surface preservation of the visceral surface of the ribcage for each individual was also scored using the grading system for erosion/abrasion to human bone proposed by McKinley (2004), who provides detailed descriptions and images of each grade. This system consists of seven grades from Grade 0 (no modifications to the bone surface) to Grade 5+ (extensive erosion of the bone surface). However, no individual presented a cortical surface preservation score of above four. The mean fragmentation score and cortical preservation score for each site was produced by calculating the average score from all individual skeletons within each site (see above).

**Table 1:** Rib cage fragmentation scores and their descriptions

Score	Description
None (0)	No fragmentation except very minor “chipping” at peripheral and vulnerable areas
Minor (1)	Fragmentation consists of large fragments, easily pieced back together; less than 25% of each element is affected
Moderate (2)	Between 25 and 75% of each element is fragmented; pieces may range in size; the morphology of the elements may still be recognized when pieces are aligned together
Severe (3)	Fragmentation is extreme, with small, often unidentifiable fragments consisting of more than 75% of each element

### 2.3 Recording true prevalence

Where possible and accounting for preservation, all available ribs from each individual were assigned a side (left or right) and seriated (1–12). Seriation was based on morphological variations between the ribs (Cirillo & Henneberg, 2012; Dudar, 1993; Mann, 1993; Scheuer & Black, 2000, pp. 232–235). In cases of poor preservation and/or fragmentation, attempts were made to identify as many fragments as possible to a side and region of the rib cage and, when it was not possible to seriate, to allocate them to the most likely position within the rib cage region: “upper” (ribs 1–3), “upper‐middle” (ribs 4–6), “lower‐middle” (ribs 7–9), or ‘lower’ (ribs 10–12). In cases where identification was particularly problematic, a category of “upper” (ribs 1–3), “middle” (ribs 4–9), or “lower” (ribs 10–12) was allocated (after Roberts et al., 1994). Ribs 1–3 and 10–12 are considered the most distinctive and are relatively easy to identify and, therefore, retain their original groupings. General osteology textbooks provide methods for identifying ribs 1, 2, 11, and 12 (Scheuer & Black, 2000, pp. 232–233; White, Black, & Folkens, 2012, p. 153, 156). Morphological descriptions of ribs 3 and 10 are provided by Scheuer and Black (2000, p. 235). However, seriation of the middle ribs (ribs 4–9) can be difficult, but the grouping of these ribs into “middle”, or “upper‐middle” and “lower‐middle” can accommodate for observers with inexperience in rib seriation.

**Figure 3:**
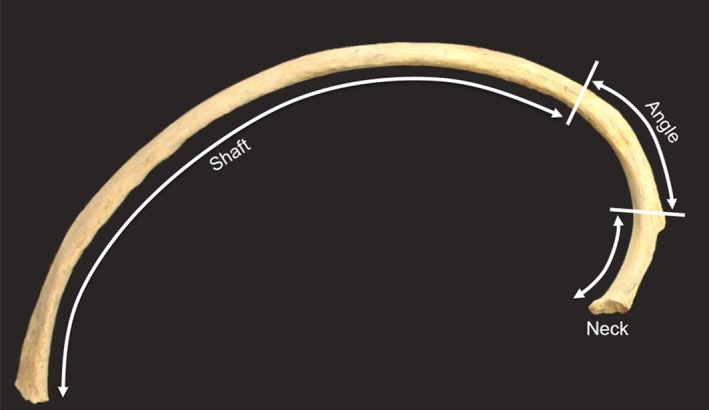
The three sections of the rib recorded separately in this study. (Image courtesy of the Trustees of The British Museum)

The presence or absence of three separate sections of the rib were also recorded (neck, angle, and shaft—Figure 3). Preservation often varied across these sections, with certain sections surviving in a better condition than others. Hence, true prevalence was calculated for each individual section. The “transition zones” of these sections were identified using specific morphological markers. The neck was considered to consist of the region of the rib from the costovertebral facet to the tubercle. The angle consisted of the section from the tubercle to the furthest extent of the angle, usually demarcated by a roughened line running supero‐inferiorly and sometimes referred to as the oblique line. The shaft consisted of the remaining body of the rib to the sternal end. These sections correspond to the “zones” recommended for recording ribs, according to the “zonation method” for fragmented human remains (Knüsel & Outram, 2004). The presence of at least 50% of each section was required for it to be recorded as present. Small fragments that could not be sided or seriated were not recorded. This represented a very small proportion of the total rib fragments observed. If two or more separate fragments (in particular for the shaft) could not be conclusively identified as belonging to separate ribs, these were not recorded to avoid inaccuracy in recording the frequency of sections. It is hoped that the detailed recording approach proposed here will help to investigate the cause of IPR in past populations, particularly if, as recommended by Waldron (2009, p. 117), the location of the pathological change and the affected ribs can be identified securely and linked to the surface markings of the lobes of the lungs and the pleura.

All available sections of each rib were analyzed macroscopically with the aid of a hand lens (x12 magnification) for pathological changes to the inner rib surfaces. The presence or absence of pathological changes, the section of the rib affected, the type of bone activity (lytic or blastic), and whether the lesions were active, remodeling, or remodeled were recorded. To test for significant differences between groups a two‐tailed Chi‐square test (*χ* ^2^), using either one (comparison between two groups) or two (comparison between three groups) degrees of freedom, was applied. A *p*‐value of less than 0.5 was used to indicate a statistically significant difference between groups.

### 2.4 Differentiating bony changes on the ribs

**Figure 4:**
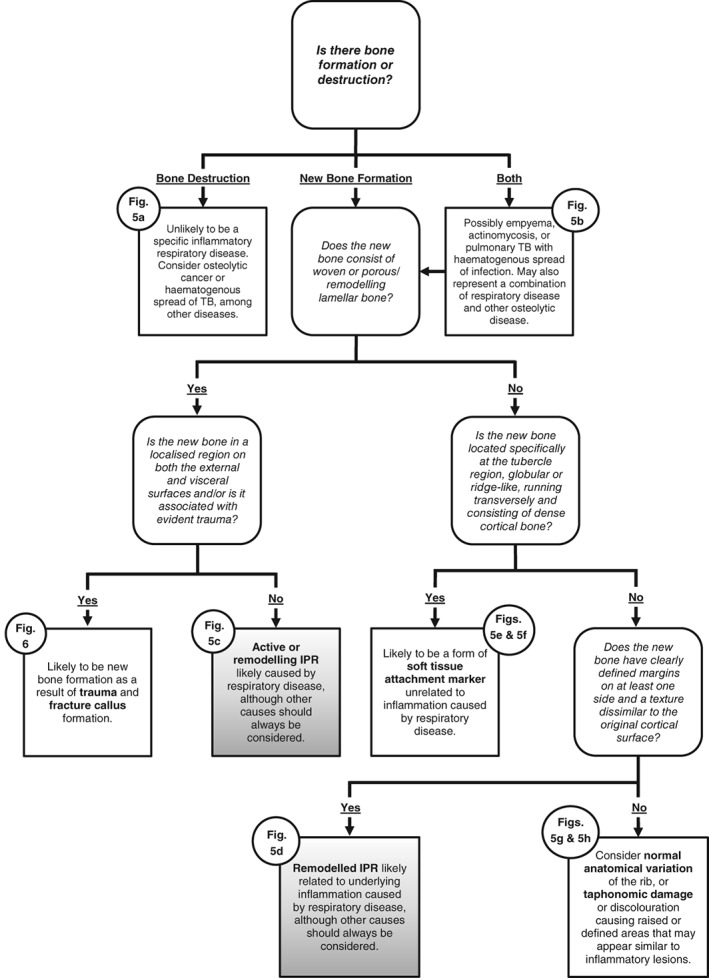
Flowchart of diagnostic criteria for identifying IPR likely related to respiratory disease (gray boxes) and for differentiating them from other bony changes to the visceral surfaces of the ribs

**Figure 5:**
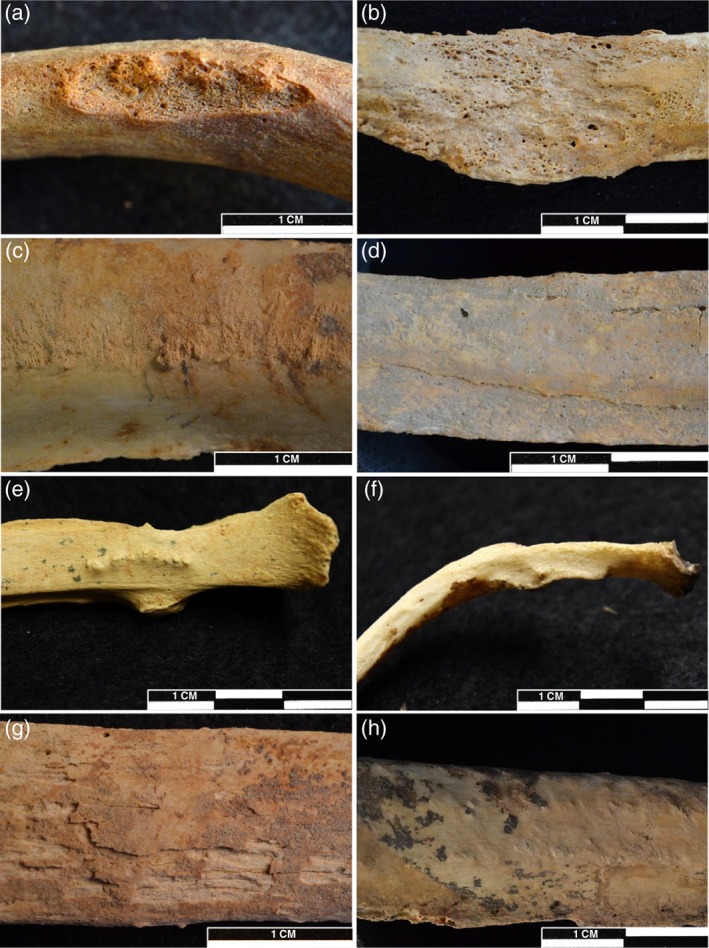
Examples of bony changes to the visceral surfaces of the ribs found on the skeletons analyzed in this study. They represent the range of pathological changes encountered in this research and match descriptions and examples found in the palaeopathological literature (Matos & Santos, 2006; Roberts et al., 1994; Santos & Roberts, 2006) (Images courtesy of the Trustees of The British Museum) (a) Destructive lesion to the superior border of the shaft, with minimal new bone formation (*Right 9th rib*). Site 3‐J‐23, Grave 59. (b) Mixed reaction on the shaft, with destruction of the cortical bone surface and active new bone formation (Left 9th rib). Site 3‐J‐18, Skeleton 4,361. (c) Subtle woven bone formation on the shaft (Right 7th rib). Site 3‐J‐23, Grave 55. (d) Remodeled lamellar bone formation on the shaft, with clearly defined margins (Left 10th rib). Site 3‐J‐23, Grave 132. (e) Globular bone formation running transversely at the tubercle region (Right 6th rib). Site Gabati, Skeleton 41–929. (f) Ridge‐like bone formation, confluent with the original cortical surface, running transversely at the neck and tubercle region (Right 4th rib). Site 3‐J‐23, Skeleton 67B. (g) Taphonomic lamination of the cortical surface, which can be mistaken for remodeled lamellar bone formation (Left upper‐middle rib). Site 3‐J‐18, Skeleton 4,188. (h) Irregular and slightly nodular surface morphology (Right lower‐middle rib). Site 3‐J‐23, Skeleton Grave 39

**Figure 6:**
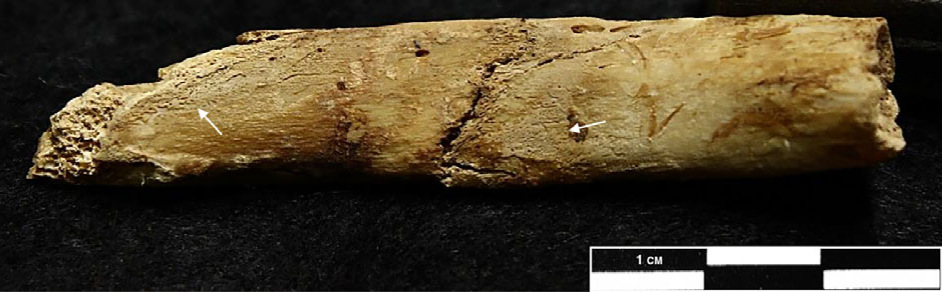
An example of subtle woven bone formation on the visceral surface of the rib (arrows), as a result of fracture (left 9th rib). This type of bone formation on the rib could be mistaken for IPR caused by lower respiratory tract disease. However, new bone formation at the margins of the break and within the trabecular bone indicates the early stages of the formation of a fracture callus. Site 3‐J‐23, grave 126 (Image courtesy of the Trustees of The British Museum)

A lack of standard diagnostic criteria or a clear definition of the type of bone changes occurring on the visceral surfaces of the ribs in previous studies suggests that there are limitations when interpreting such pathological changes (Naples & Rothschild, 2011). There are several types of bony changes that can occur but not all of them necessarily relate to IPR caused by lower respiratory tract disease (see below). It is unclear which bioarchaeological studies of lower respiratory tract disease have considered unusual types of bony changes on the visceral surfaces of the ribs to represent respiratory disease, and this may affect comparability between studies. Therefore, diagnostic criteria for identifying the bony changes most likely to be specifically related to lower respiratory tract disease are proposed here, with the aim of increasing consistency within and between future studies. To aid in identification and differential diagnosis, various bony changes are described in detail, including a diagnostic flow chart (Figure 4) with accompanying images (Figures 5 and 6).

#### 2.4.1 Inflammatory periosteal reaction (on the visceral surface)

Only diffuse (of irregular location, affecting the surface of the bone in a continuous or widespread distribution) new bone formation on the visceral surface was considered to be evidence for IPR and, therefore, for inflammatory processes related to lower respiratory tract disease. Considering the surrounding anatomical structures, diffuse IPR on the visceral surfaces of the ribs is likely to be evidence of respiratory‐related inflammatory processes, although other conditions such as trauma or a chest wall tumor may be responsible (Waldron, 2009, p. 117).

Diffuse woven bone and porous remodeling lamellar bone are recognizable as layers of new bone formed on top of the original cortical surface that appear irregular, disorganized or waxy/porous (Ortner, 2012; Weston, 2008, 2012) (Figure 5c). Matos and Santos (2006, Table 2) provide a useful table for macroscopically differentiating woven and lamellar bone. While woven bone appears extremely porous, with a heterogenous unstructured surface and irregular margins, lamellar bone is compact and porosity can be sparse or non‐existent. However, completely remodeled lamellar bone can often be confluent with the original surface and difficult to differentiate from normal anatomical variation or taphonomic lamination/discoloration. To reduce false positive recording, remodeled lamellar IPR should only be considered present if the new bone forms a distinct layer on top of the original cortical surface, with clearly defined margins and/or a surface texture dissimilar to the original cortical bone (Figure 5d). While these criteria may exclude some examples of small thin regions of highly remodeled IPR that are difficult to differentiate from the original cortical surface, they do reduce false positive data and will increase consistency within and between studies. New bone formation in response to trauma may have the same appearance as rib IPR caused by lower respiratory tract disease (Figure 6). It is important, therefore, to look for evidence of remodeling fracture margins or localized new bone formation on both the visceral and outer surfaces of the rib.

**Table 2:** True prevalence rates of rib IPR presented by rib number for the sites of 3‐J‐23, 3‐Q‐33, and 4‐L‐2. Frequencies of rib sections displaying IPR are presented in brackets

Site	Side	Section	Rib number												Total		
			1	2	3	4	5	6	7	8	9	10	11	12	Section	Side	Rib cage
3‐J‐23	Right	Neck	3.2% (6/62)	11.1% (7/63)	15.6% (10/64)	14.5% (9/62)	16.1% (10/62)	12.9% (8/62)	16.1% (10/62)	12.9% (8/62)	11.3% (7/62)	11.1% (7/63)	6.3% (4/64)	3.4% (2/58)	11.3% (84/746)	8.4% (186/2227)	8.5% (381/4502)
		Angle	3.1% (2/64)	9.4% (6/64)	12.5% (8/64)	12.9% (8/62)	12.9% (8/62)	9.7% (6/62)	6.5% (4/62)	9.7% (6/62)	8.1% (5/62)	6.3% (4/64)	4.6% (3/65)	1.8% (1/56)	8.1% (61/749)		
		Shaft	4.7% (3/64)	8.2% (5/61)	11.5% (7/61)	8.2% (5/61)	6.5% (4/62)	8.3% (5/60)	4.8% (3/62)	6.7% (4/60)	3.3% (2/60)	1.6% (1/62)	1.6% (1/63)	1.8% (1/56)	5.6% (41/732)		
	Left	Neck	0.0% (0/65)	9.2% (6/65)	15.9% (10/63)	19.4% (12/62)	17.5% (11/63)	14.3% (9/63)	14.5% (9/62)	11.1% (7/63)	9.5% (6/63)	6.1% (4/66)	3.1% (2/65)	1.7% (1/59)	10.1% (77/759)	8.6% (195/2275)	
		Angle	0.0% (0/65)	4.6% (3/65)	10.8% (7/65)	12.7% (8/63)	15.9% (10/63)	12.7% (8/63)	14.3% (9/63)	14.3% (9/63)	9.5% (6/63)	9.1% (6/66)	4.5% (3/66)	1.7% (1/59)	9.2% (70/764)		
		Shaft	0.0% (0/65)	4.8% (3/62)	9.5% (6/63)	11.3% (7/62)	6.3% (4/63)	7.9% (5/63)	6.6% (4/61)	6.5% (4/62)	6.3% (4/63)	7.8% (5/64)	7.6% (5/66)	1.7% (1/58)	6.4% (48/752)		
3‐Q‐33	Right	Neck	0.0% (0/18)	5.9% (1/17)	5.3% (1/19)	5.9% (1/17)	5.6% (1/18)	11.1% (2/18)	11.1% (2/18)	11.8% (2/17)	11.8% (2/17)	11.8% (2/17)	5.9% (1/17)	5.9% (1/17)	7.6% (16/210)	3.0% (19/626)	4.4% (55/1245)
		Angle	0.0% (0/17)	0.0% (0/19)	0.0% (0/20)	0.0% (0/18)	0.0% (0/19)	0.0% (0/17)	0.0% (0/18)	0.0% (0/19)	5.6% (1/18)	0.0% (0/19)	0.0% (0/19)	0.0% (0/16)	0.5% (1/219)		
		Shaft	0.0% (0/15)	0.0% (0/17)	0.0% (0/14)	0.0% (0/18)	0.0% (0/17)	0.0% (0/14)	0.0% (0/18)	0.0% (0/16)	5.9% (1/17)	0.0% (0/17)	5.3% (1/19)	0.0% (0/15)	1.0% (2/197)		
	Left	Neck	12.5% (2/16)	0.0% (0/18)	7.7% (1/13)	0.0% (0/17)	5.9% (1/17)	5.6% (1/18)	15.8% (3/19)	16.7% (3/18)	21.1% (4/19)	23.5% (4/17)	12.5% (2/16)	6.7% (1/15)	10.8% (22/203)	5.8% (36/619)	
		Angle	5.3% (1/19)	5.0% (1/20)	0.0% (0/20)	5.3% (1/19)	5.9% (1/17)	0.0% (0/19)	5.3% (1/19)	0.0% (0/18)	5.6% (1/18)	0.0% (0/18)	0.0% (0/19)	0.0% (0/17)	2.7% (6/223)		
		Shaft	5.6% (1/18)	0.0% (0/14)	13.3% (2/15)	5.9% (1/17)	0.0% (0/13)	5.6% (1/18)	6.7% (1/15)	0.0% (0/17)	0.0% (0/17)	5.6% (1/18)	6.3% (1/16)	0.0% (0/15)	4.1% (8/193)		
4‐L‐2	Right	Neck	0.0% (0/10)	0.0% (0/16)	0.0% (0/14)	0.0% (0/7)	12.5% (1/8)	0.0% (0/7)	0.0% (0/8)	16.7% (1/6)	12.5% (1/8)	23.1% (3/13)	0.0% (0/18)	0.0% (0/17)	4.5% (6/132)	6.5% (21/325)	4.2% (27/647)
		Angle	7.1% (1/14)	7.1% (1/14)	8.3% (1/12)	10.0% (1/10)	0.0% (0/9)	0.0% (0/7)	0.0% (0/8)	14.3% (1/7)	0.0% (0/7)	7.7% (1/13)	7.1% (1/14)	7.1% (1/14)	6.2% (8/129)		
		Shaft	0.0% (0/10)	0.0% (0/5)	20.0% (1/5)	28.6% (2/7)	0.0% (0/4)	33.3% (1/3)	25.0% (1/4)	0.0% (0/4)	0.0% (0/3)	16.7% (1/6)	0.0% (0/6)	14.3% (1/7)	10.9% (7/64)		
	Left	Neck	0.0% (0/8)	0.0% (0/16)	0.0% (0/15)	0.0% (0/9)	0.0% (0/7)	0.0% (0/5)	16.7% (1/6)	0.0% (0/6)	0.0% (0/6)	10.5% (2/19)	5.3% (1/19)	0.0% (0/15)	3.1% (4/131)	1.9% (6/322)	
		Angle	0.0% (0/14)	0.0% (0/13)	0.0% (0/15)	0.0% (0/9)	0.0% (0/7)	0.0% (0/5)	0.0% (0/6)	0.0% (0/5)	0.0% (0/6)	5.9% (1/17)	0.0% (0/15)	6.7% (1/15)	1.6% (2/127)		
		Shaft	0.0% (0/12)	0.0% (0/4)	0.0% (0/4)	0.0% (0/5)	0.0% (0/4)	0.0% (0/2)	0.0% (0/3)	0.0% (0/3)	0.0% (0/3)	0.0% (0/5)	0.0% (0/8)	0.0% (0/15)	0.0% (0/64)		

#### 2.4.2 Irregular surface morphology (on the shaft)

The surfaces of the shafts of the ribs can often be irregular (Figure 5h) and striated or nodular surfaces may indicate initial or healed subtle inflammatory lesions (Nicklisch et al., 2012). However, these surface irregularities are not directly indicative of diffuse inflammatory periosteal new bone. The attachment sites of the intercostal muscles at the superior and inferior margins of the ribs often extend onto the visceral surface itself (Naples & Rothschild, 2011), and it is not known to what extent these attachments affect variability in surface morphology. Irregular surface morphology was, therefore, not considered to be evidence of IPR.

#### 2.4.3 Transverse bone formation

Dense globular or ridge‐like new bone running transversely along the visceral surface in the neck and tubercle regions occurred frequently in skeletal remains from the sites analyzed in this study (Figure 5e, f). Variable in size, this unusual bone formation consists of dense cortical bone, often confluent with the original cortical surface, and is reminiscent of osteoblastic bone changes related to entheseal changes (bone forming at tendon or ligament attachment sites) or exostosis (a benign bony growth on the surface of a bone) (Henderson, Mariotti, Pany‐Kucera, Villotte, & Wilczak, 2016; Ponce, 2010; Resnick, 2002, Chapter 76).

Due to the specific location, characteristics of the bone, and transverse organization, these lesions did not appear to be consistent with reaction to diffuse inflammation of the region and, as yet, there is no clinical evidence that inflammation as the result of lower respiratory tract disease causes this specific type of bone formation. For these reasons, transverse bone formation at the neck and tubercle regions was not considered when recording IPR of the visceral rib surfaces. The consistent position of this type of bone formation at the neck and tubercle regions suggests that it is related to specific soft‐tissue attachments in this area. This bone change may be evidence for the origin or insertion points of subcostal muscles or the result of extreme strain around the costo‐transverse ligament, and subsequent trauma or micro‐tearing of the endothoracic fascia or localized soft tissue. Similar changes have been noted by Lambert (2002) in skeletons from Cowboy Wash, southwestern Colorado, USA, and were suggested to be caused by a mechanical stressor. However, further research into the formation of this unusual bone formation is required, such as the potential for it to be related to a chronic cough.

#### 2.4.4 Destructive or mixed reaction lesions on any region of the rib

Lytic lesions with minimal to no new bone formation (Figure 5a) were not considered as direct evidence of inflammation related to respiratory disease. These lesions may be caused by pathological processes related to cancer, hematogenous spread of extrapulmonary tuberculosis, or other conditions leading to bone destruction (Guttentag & Salwen, 1999; Jeung et al., 1999). Extension of destructive lesions from the spine to the heads and necks of the ribs in tuberculosis has been noted (Capasso & Di Tota, 1999; Mays, Fysh, & Taylor*,* 2002; Ortner, 2003, p. 246), but is likely to be the indirect impact of spinal tuberculosis rather than specific infection of the lower respiratory tract by *Mycobacterium tuberculosis*. While pulmonary tuberculosis may also cause destructive lesions on the ribs, skeletal involvement in this form of the disease is uncommon (Asnis & Niegowska, 1997; Talbot et al., 2007). Tuberculosis can be contracted in other ways, such as from the ingestion of infected foods, rather than through infection of the respiratory tract. Therefore, it is difficult to distinguish destructive lesions caused by pulmonary tuberculosis from other disease processes in archeological skeletons, such as extra‐pulmonary tuberculosis or neoplastic disease, without evidence in other regions of the skeleton.

A mix of bone formation and destruction on the visceral surfaces of the ribs (Figure 5b) has been linked to respiratory diseases such as actinomycosis, tuberculosis, and pneumonia, which can cause pleural disease, empyema and subsequent osteomyelitis of the ribs (Guttentag & Salwen, 1999; Jeung et al., 1999; Madeo, Patel, Gebre, & Ahmed, 2013). Additionally, mixed bone reaction may also be the result of a combination of IPR, due to lower respiratory tract disease, and another osteolytic bone disease. In this study, if diffuse IPR was present alongside destructive lesions, this was considered to be evidence for respiratory disease. However, it should also be noted that a mixed reaction from neoplastic disease or osteomyelitis, as a result of chest wall trauma, can also be considered as a cause, and it is essential to record pathological changes across the entire skeleton in attempting differential diagnoses and to diagnose a specific disease. Neoplastic disease can be hard to differentiate from other diseases (Ragsdale, Campbell, & Kirkpatrick, 2018) and often requires additional methods of identification, such as radiography (Binder, Roberts, Spencer, Antoine, & Cartwright, 2014). However, mixed reaction neoplastic disease, such as metastatic carcinoma, may manifest in multiple bones within one individual. Observing such lesions beyond the rib cage may help to exclude respiratory disease as the cause of rib IPR in these instances (Binder et al., 2014; Lieverse, Temple, & Bazaliiskii, 2014).

## 3 RESULTS

True prevalence rates were produced for all the sites according to each section of the rib (neck, angle, and shaft) by rib number (1–12) and side (Table 2), and by rib cage region and side (Table 3). The prevalence rates for sites 3‐Q‐33 and 3‐J‐23 showed little to no variation between the section of rib affected, side of rib affected, and the whole rib cage when presented either by rib cage region or by rib number, with the greatest percentage difference being only 0.4% (3‐Q‐33, left shaft total prevalence). The more fragmented site (4‐L‐2) displayed a slight increase in prevalence when data were presented according to rib cage region ‐ both left rib shaft total prevalence and right angle total prevalence increased by 1.4%. However, presenting prevalence by rib cage region also increased the number of rib sections included in the sample by 236 for 4‐L‐2.

**Table 3:** True prevalence rates of rib IPR presented by rib cage region for the sites of 3‐J‐23, 3‐Q‐33, and 4‐L‐2. Frequencies of rib sections displaying IPR are presented in brackets

Site	Side	Section	Rib cage region					Total		
			Upper	Upper‐Middle	Middle	Lower‐Middle	Lower	Section	Side	Rib cage
3‐J‐23	Right	Neck	10.1% (19/189)	14.0% (27/193)	13.5% (52/384)	13.2% (25/189)	7.0% (13/185)	11.1% (84/758)	8.4% (191/2266)	8.5% (386/4568)
		Angle	8.3% (16/192)	12.4% (24/193)	10.4% (40/386)	8.4% (16/191)	4.3% (8/186)	8.4% (64/764)		
		Shaft	8.1% (15/186)	8.1% (15/185)	6.7% (25/374)	4.8% (9/188)	1.6% (3/184)	5.8% (43/744)		
	Left	Neck	8.2% (16/194)	16.6% (32/193)	14.0% (54/387)	11.5% (22/192)	3.7% (7/190)	10.0% (77/771)	8.5% (195/2302)	
		Angle	5.1% (10/195)	13.4% (26/194)	12.8% (50/390)	12.4% (24/194)	5.2% (10/191)	9.0% (70/776)		
		Shaft	4.8% (9/189)	8.5% (16/189)	7.4% (28/378)	6.3% (12/189)	5.9% (11/188)	6.4% (48/755)		
3‐Q‐33	Right	Neck	3.7% (2/54)	7.4% (4/54)	9.1% (10/110)	11.3% (6/53)	7.7% (4/52)	7.4% (16/216)	3.0% (19/642)	4.4% (56/1274)
		Angle	0.0% (0/56)	0.0% (0/55)	0.9% (1/113)	1.8% (1/55)	0.0% (0/55)	0.4% (1/224)		
		Shaft	0.0% (0/46)	0.0% (0/51)	1.0% (1/104)	2.0% (1/51)	1.9% (1/52)	1.0% (2/202)		
	Left	Neck	6.4% (3/47)	3.8% (2/53)	11.0% (12/109)	16.7% (10/60)	14.0% (7/50)	10.7% (22/206)	5.9% (37/632)	
		Angle	3.4% (2/59)	3.6% (2/56)	3.5% (4/114)	3.6% (2/56)	0.0% (0/55)	2.6% (6/228)		
		Shaft	6.4% (3/47)	4.1% (2/49)	4.0% (4/100)	2.0% (1/49)	3.9% (2/51)	4.5% (9/198)		
4‐L‐2	Right	Neck	0.0% (0/41)	2.6% (1/38)	6.3% (7/112)	8.3% (3/36)	6.0% (3/50)	4.9% (10/203)	6.8% (30/441)	4.5% (40/883)
		Angle	7.3% (3/41)	5.4% (2/37)	7.8% (7/90)	6.3% (2/32)	7.3% (3/41)	7.6% (13/172)		
		Shaft	5.0% (1/20)	21.4% (3/14)	14.8% (4/27)	9.1% (1/11)	10.5% (2/19)	10.6% (7/66)		
	Left	Neck	0.0% (0/36)	0.0% (0/36)	1.9% (2/106)	3.0% (1/33)	5.7% (3/53)	2.6% (5/195)	2.3% (10/442)	
		Angle	0.0% (0/40)	0.0% (0/33)	2.2% (2/89)	0.0% (0/29)	4.2% (2/48)	2.3% (4/177)		
		Shaft	0.0% (0/18)	0.0% (0/13)	3.6% (1/28)	0.0% (0/12)	0.0% (0/24)	1.4% (1/70)		

**Figure 7:**
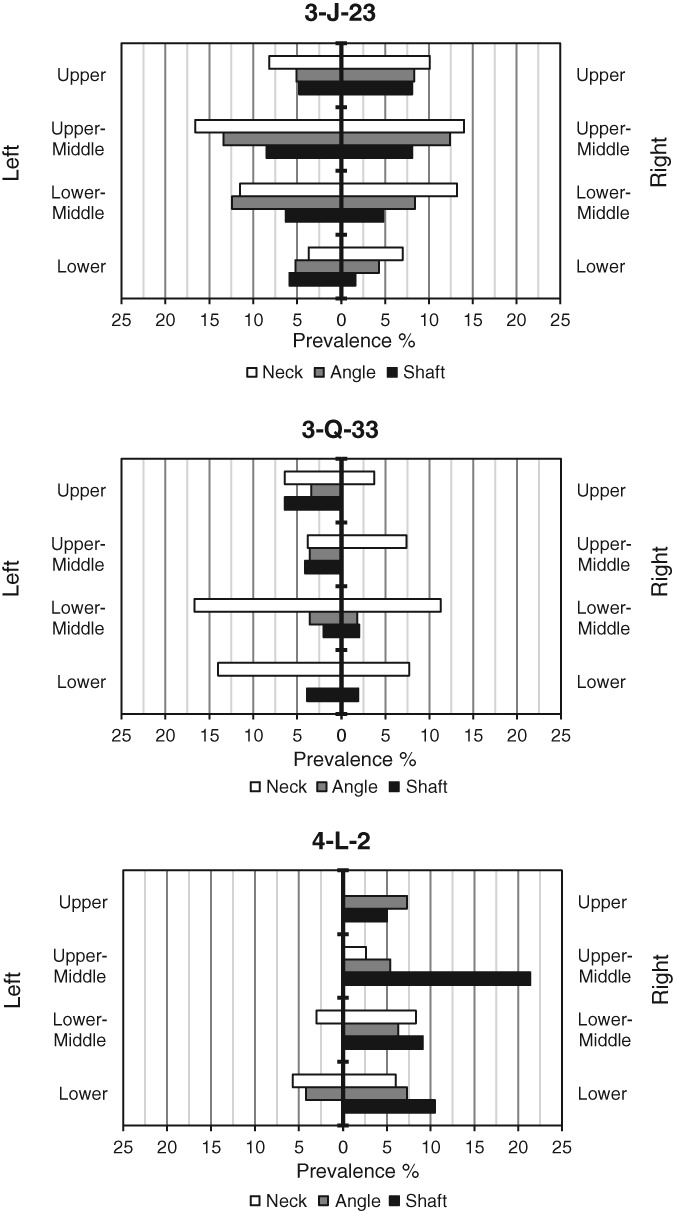
True prevalence rates for sites 3‐J‐23 (top), 3‐Q‐33 (middle), and 4‐L‐2 (bottom), presented by side, rib cage region, and section affected

Using graphs (Figure 7), it is possible to visually compare distribution patterns of IPR between the sites. To compare these sites, true prevalence is presented according to rib cage regions affected (upper, upper‐middle, lower‐middle, and lower). It is evident that the distribution of IPR within the rib cage, by side and section affected, varies among the three sites. While prevalence on either side of the rib cage for 3‐J‐23 is relatively equal, 3‐Q‐33 displays a statistically significant 2.8% increase in prevalence on the left side (*χ* ^2^ [1] = 5.6994; *p* = 0.017), while 4‐L‐2 has a statistically significant increase of 4.6% in prevalence on the right side (*χ* ^2^ (1) = 10.522; *p* = <0.01). When the total for both the right and left sides of each section type is calculated, 3‐Q‐33 also displays a statistically significantly higher prevalence in the neck section than in other sections (*χ* ^2^ (2) = 43.86; *p* = <0.01).

Crude prevalence rates for 3‐J‐23, 3‐Q‐33, and 4‐L‐2 are 43.9% (29/66), 45.0% (9/20), and 23.1% (6/26), respectively. However, true prevalence when calculated by all sections within the entire sample did not correlate with crude prevalence. For example, although 3‐Q‐33 displayed the highest crude prevalence rate, the total true prevalence for the entire sample of 4.4% is comparable to 4‐L‐2 (4.5% by rib cage region), a site which displayed a considerably lower crude prevalence.

## 4 DISCUSSION

Results indicate that for skeletons from sites which produce greater fragmentation of the ribs, increasing the difficulty of rib seriation, the presentation of true prevalence rates for IPR by rib cage region rather than by specific rib number can greatly increase the sample size, as seen in the more fragmented site 4‐L‐2. The use of rib cage regions also allows for observers with inexperience in rib seriation and provides a mechanism for comparison between skeletons from sites that are differentially preserved. The lack of variation in the total true prevalence rate for IPR, when presented by rib number or rib cage region in the well‐preserved sites of 3‐J‐23 and 3‐Q‐33, suggests that the presentation of data by rib cage region in skeletons from well preserved sites for the purposes of comparison with more fragmented sites will not compromise the accuracy of the results. Therefore, it is desirable to present data by rib cage region rather than by rib number. However, small subtleties in the distribution of IPR across the rib cage, which may be related to specific anatomical structures and the formation of IPR, may be lost. In effect, for sites where fragmentation is minimal, presentation by rib number in addition to rib cage region may also be advantageous. Any data presented by rib number in these sites can also be easily converted into rib cage region, allowing comparison with more fragmented skeletal remains.

Despite the lower crude prevalence rate for 4‐L‐2, true prevalence rates for certain sections of the right side of the rib cage, by rib number, are comparable to 3‐J‐23. They also exceed true prevalence for the right side in skeletons from 3‐Q‐33, although this site has the highest crude prevalence. The smaller sample size of 4‐L‐2, when compared to the other sites, may play a part in this high prevalence. However, it is possible that the total prevalence rate of IPR within the site of 4‐L‐2 was not lower than for the other sites, but that fragmentation of the skeletons reduced the number of observable ribs per individual, and thus compromised the calculation of crude prevalence.

Nevertheless, crude prevalence should ideally be presented alongside true prevalence for this pathological condition. The number of ribs displaying IPR in skeletons within a site is not a direct indication of the number of individuals who may have been affected by respiratory disease. This is because individuals may display varying degrees of rib cage involvement, from one rib to the entire rib cage. Differences between true and crude prevalence rates among sites may be the result of the involvement of a greater distribution of rib IPR across the rib cage in some individuals (i.e., IPR in the rib cages of certain skeletons may have involved multiple sites, increasing the true prevalence). Instead, the two methods of calculating prevalence can perform different roles in the analysis of respiratory disease in people buried within a site. While crude prevalence can aid in understanding the potential extent that respiratory disease affected the population (although only realistically for well‐preserved sites), true prevalence rates can provide a more accurate understanding of the distribution of IPR throughout the rib cages of individuals.

It has been suggested that the region of the rib cage affected may provide some insight to the specific disease causing the IPR (Santos & Roberts, 2006; Waldron, 2009) and greater involvement of certain regions of the rib cage has been noted by previous authors (Kelley et al., 1994; Lambert, 2002; Nicklisch et al., 2012). Additionally, studies of rib IPR in skeletons with known biographical information have identified different IPR distributions in association with a variety of diseases listed as cause of death (although inaccuracies in the recorded cause of death may be present in some of the biographical data). Greater involvement of the head and neck, in particular of the middle region of the rib cage, has been recorded in individuals who died from tuberculosis (Matos & Santos, 2006; Roberts et al., 1994; Santos & Roberts, 2006) and a higher likelihood of sternal end involvement has been found in individuals who died from non‐tuberculosis pulmonary diseases (Matos & Santos, 2006). In addition, unilateral lesions in individuals dying from tuberculosis have been found to more likely affect the left ribs with a ratio of approximately 2:1 (Kelley & Micozzi, 1984; Santos & Roberts, 2006). Clinically, it has been suggested that greater involvement of the left lung in tuberculosis may be due to the proximity of, and space limitation caused by, the aorta, which makes the left lung more prone to obstruction by enlarged lymph nodes (Ashour, 1995). At 3‐Q‐33, a significantly higher true prevalence rate was found on the left side of the rib cage and at the neck section, while at 4‐L‐2, the right side displayed a significantly higher prevalence. This may indicate potential differences in the etiology of rib IPR in individuals at these sites.

It has been hypothesized that the IPR distribution on the ribs in people with lobar pneumonia may correspond to the anatomical position of the specific lobe affected (Waldron, 2009, p.117). In examples of very complete rib cages, such as in skeletons from 3‐J‐23 or 3‐Q‐33, it may be possible to compare prevalence by rib number to anatomical position of the lobes. Additionally, it has been found that individuals suffering from peritonitis (inflammation of the peritoneum lining the abdominal cavity) displayed IPR on the lower ribs (Santos & Roberts, 2006), which corresponds to spread of inflammation from the peritoneum to the lower pleural cavity. Therefore, a careful consideration of the anatomical structures underlying the rib cage in relation to IPR distribution should contribute to diagnosing specific disease processes. The use of statistical analysis of prevalence data can further highlight significant differences between different rib sections, rib cage sides or regions, and between skeletons from different sites.

There are still limitations to recording IPR, most importantly that of poor cortical bone preservation due to taphonomic destruction, hindering the observation of IPR on the ribs, for which this method does not account. In this study, fragmentation and cortical preservation scores show similar trends within sites, suggesting that high levels of fragmentation at a site may be accompanied by high levels of cortical surface damage. Overcoming this problem requires researchers to be transparent in reporting the cortical surface preservation of ribs from skeletons from their sites, so that the suitability of resulting data for comparison to other sites can be considered. Additionally, the requirement that 50% or more of the rib section must be present for it to be recorded may also cause slight underestimations in true prevalence. In the case of the rib shaft section, the absence of 50% constitutes a substantial unobservable surface area, which may, if preserved, have presented evidence for IPR. It should also be noted that the greater inclusion of poorly preserved skeletons will also increase the number of individuals for whom age and sex cannot be accurately estimated. This will limit comparison among sites when investigating the prevalence of rib IPR in different age or sex categories.

Nevertheless, the method presented here is the first detailed scheme for recording rib lesions in archeological populations with variable preservation and, if used by researchers, will improve standardization in recording and presenting true prevalence rates for rib IPR. Using this method, and with the incorporation of clinical studies of lower respiratory tract disease in the interpretation of resulting data, it is possible to start to further investigate the potential to identify specific disease processes affecting different regions of the rib cage in skeletons from archeological sites. Furthermore, when studying larger numbers of individuals, the analysis of the distribution of IPR in different groups, such as between the sexes or ages, may help to identify exposure to different pathogens as a result of risk factors within past paleoenvironments, such as through work related poor air quality. Additionally, this method will be particularly useful when applied to comingled remains, for which crude IPR prevalence data may be difficult to obtain, and can be adapted for the use of recording other pathological changes in ribs, such as rib fracture prevalence and its distribution within the rib cage.

Returning to the aim of this study, a detailed method for recording IPR on the visceral surfaces of ribs has been achieved and the diagnostic “flow chart” method presented here has been used to identify IPR related to lower respiratory tract disease and differentiate it from other bony changes. This method has provided consistency for recording and producing true prevalence rates for skeletons, even for severely fragmented remains, from different regions and time periods, in this case from the Middle Nile Valley. The approach proposed here will enable researchers to apply more rigor in their differential diagnosis when studying individual skeletons and allow for like‐with‐like comparisons when recording large populations with differing preservation.
